# The time-course of cancer cachexia onset reveals biphasic transcriptional disruptions in female skeletal muscle distinct from males

**DOI:** 10.1186/s12864-023-09462-7

**Published:** 2023-07-04

**Authors:** Francielly Morena da Silva, Seongkyun Lim, Ana Regina Cabrera, Eleanor R. Schrems, Ronald G. Jones, Megan E. Rosa-Caldwell, Tyrone A. Washington, Kevin A. Murach, Nicholas P. Greene

**Affiliations:** 1 Cachexia Research Laboratory, Exercise Science Research Center, Department of Health, Human Performance and Recreation, University of Arkansas, Fayetteville, AR USA; 2 Exercise Muscle Biology Laboratory, Exercise Science Research Center, Department of Health, Human Performance and Recreation, University of Arkansas, Fayetteville, AR USA; 3 Molecular Muscle Mass Regulation Laboratory, Exercise Science Research Center, Department of Health, Human Performance and Recreation, University of Arkansas, Fayetteville, AR USA

**Keywords:** Lewis lung carcinoma, Biological sex dimorphism, RNA-sequencing, Type-II interferon, Cancer cachexia, Mitocarta

## Abstract

**Background:**

Cancer-cachexia (CC) is a debilitating condition affecting up to 80% of cancer patients and contributing to 40% of cancer-related deaths. While evidence suggests biological sex differences in the development of CC, assessments of the female transcriptome in CC are lacking, and direct comparisons between sexes are scarce. This study aimed to define the time course of Lewis lung carcinoma (LLC)-induced CC in females using transcriptomics, while directly comparing biological sex differences.

**Results:**

We found the global gene expression of the gastrocnemius muscle of female mice revealed biphasic transcriptomic alterations, with one at 1 week following tumor allograft and another during the later stages of cachexia development. The early phase was associated with the upregulation of extracellular-matrix pathways, while the later phase was characterized by the downregulation of oxidative phosphorylation, electron transport chain, and TCA cycle. When DEGs were compared to a known list of mitochondrial genes (MitoCarta), ~ 47% of these genes were differently expressed in females exhibiting global cachexia, suggesting transcriptional changes to mitochondrial gene expression happens concomitantly to functional impairments previously published. In contrast, the JAK-STAT pathway was upregulated in both the early and late stages of CC. Additionally, we observed a consistent downregulation of Type-II Interferon signaling genes in females, which was associated with protection in skeletal muscle atrophy despite systemic cachexia. Upregulation of Interferon signaling was noted in the gastrocnemius muscle of cachectic and atrophic male mice. Comparison of female tumor-bearing mice with males revealed ~ 70% of DEGs were distinct between sexes in cachectic animals, demonstrating dimorphic mechanisms of CC.

**Conclusion:**

Our findings suggest biphasic disruptions in the transcriptome of female LLC tumor-bearing mice: an early phase associated with ECM remodeling and a late phase, accompanied by the onset of systemic cachexia, affecting overall muscle energy metabolism. Notably, ~ 2/3 of DEGs in CC are biologically sex-specific, providing evidence of dimorphic mechanisms of cachexia between sexes. Downregulation of Type-II Interferon signaling genes appears specific to CC development in females, suggesting a new biological sex-specific marker of CC not reliant on the loss of muscle mass, that might represent a protective mechanism against muscle loss in CC in female mice.

**Supplementary Information:**

The online version contains supplementary material available at 10.1186/s12864-023-09462-7.

## Introduction

Cancer cachexia (CC) is a syndrome experienced by up to 80% of cancer patients [1]. Despite its first description in 1858, literature lacked a formal definition until 2011, when Fearon et al. [2] described cachexia as a multifactorial condition characterized by skeletal muscle mass loss, with or without fat mass loss, resistant to conventional nutritional support and leads to a progressive functional impairment [2, 3]. CC can lower quality of life, reduce tolerance to anti-cancer drugs, and is directly responsible for 20–40% of cancer-related deaths [1, 4, 5]. Regardless of the severity of this condition, mechanisms behind CC are not fully elucidated, and effective therapies are unavailable to cancer patients.

Even though biological sex plays an important role in health and various diseases [6–9] including CC, pre-clinical research is still male-dominant [10, 11]. Transcriptomic analysis is a robust tool for uncovering the etiology of the disease [10, 12, 13]. Yet, limited studies utilize this approach to identify transcriptomic alterations at the onset and initial development of CC [10, 12, 14–16], with fewer still considering the female biological sex [10]. Our group and others have recently shown biological sex differences during the onset and progression of CC through time-course studies [10, 17–21], including muscle mass protection noted in females with systemic cachexia [17], but not in males. For instance, CC is known to be driven by systemic inflammation with characteristic induction of inflammatory cytokines including IL-6 and TNFα, followed by a causal sequence of a catabolic shift and loss of skeletal muscle mass. Male colorectal CC has long been described as IL-6 dependent, while more recent data suggest IL-6 independence in female colorectal CC [21–24]. In addition, our laboratory reported early onset mitochondrial degeneration during the development of LLC-induced CC in males [19], while such alterations are not present until the development of cachexia in females [17]. Combined current data suggest mechanisms of CC within a type of cancer are biologically sex-dependent, however, the extent and identity of such differences remain largely unknown leaving a critical gap in the literature.

Here, we utilize RNA sequencing (RNA-seq) in a time-course fashion of tumor development to achieve the two-fold purpose of 1) understanding the transcriptomic profile of protected gastrocnemius muscle mass in female mice undergoing systemic LLC-induced CC, and 2) directly comparing the gene expression landscape between males and females during the time course of early cancer development. About two-thirds of all dysregulated genes were distinct between sexes, demonstrating unique biological sex signatures and thus likely biological sex-specific mechanisms in LLC-induced CC. Sex-specific considerations are therefore imperative for developing individualized therapeutics for improving muscle mass during cancer.

## Results

After three to four weeks of tumor growth, we observed a clear distinction in tumor size between the animals. We then reorganized the animals into two groups based on their tumor size: low tumor (LT) group, consisting of tumors weighing 1.2 g or less, and high tumor (HT) group, consisting of tumors weighing 2 g or more. This approach was based on the well-established correlation between tumor size and body weight, as well as muscle weight, as larger tumors tend to correspond with smaller muscle sizes [25–27]. Phenotypic and global expression analyses were performed on a cohort of animals from a larger study (*n* = 8/condition) [17]. Tumor-free body weight was not different between experimental groups compared to PBS. EDL mass was not different between experimental conditions. Plantaris showed ~ 11% higher mass in LT compared to HT, but no differences in cancer groups when compared to PBS control (Table1, *p* = 0.04). Soleus and tibialis anterior muscles were 19.3% and 9.3% lower in HT compared to PBS, respectively (Table 1, *p* = 0.002, and *p* = 0.04). The Gastrocnemius muscle of tumor-bearing groups was not statistically different from PBS. As for visceral organs, the liver mass of LT and HT were 23% and 43% higher than PBS (Table 1, *p* = 0.02, *p* < 0.0001). Spleen mass was higher in HT by 26.5%, 35%, 48.5%, and 44%, compared to 1wk, 2wk, LT, and PBS, respectively (*p* < 0.0001). Gonadal fat was 55.9% lower in HT compared to PBS (Table 1, *p* = 0.04). Phenotypic data demonstrate multiple hallmarks of CC in HT, confirming the induction of LLC-induced CC for the currently selected subset.

**Table 1:** Phenotypic Data of Subset. Data expressed as mean +—SEM. *P* < 0.05. Different letters indicate statistical significance (*p* < 0.05)

Group	PBS	1wk	2wk	LT	HT
Body weight (g)	18.89 ± 0.21ab	17.64 ± 0.18a	18.61 ± 0.20a	19.91 ± 0.44b	21.24 ± 0.57bc
Tumor Weight (mg)	N/A	33.85 ± 4.83a	220.2 ± 41.7ab	506.4 ± 106.4b	3078 ± 141.5c
Body Weight – Tumor Weight (g)	18.89 ± 0.21ab	17.60 ± 0.19b	18.39 ± 0.23ab	19.41 ± 0.38a	18.16 ± 0.54ab
Gastrocnemius (mg)	89.60 ± 1.17abc	85.42 ± 1.88ac	88.16 ± 1.45abc	94.63 ± 1.65b	85.58 ± 2.17c
Soleus (mg)	7.3 ± 0.18a	6.43 ± 0.18ab	6.84 ± 0.35ab	7.28 ± 0.17a	5.89 ± 0.27b
Plantaris (mg)	12.78 ± 0.43ab	12.43 ± 0.36ab	12.65 ± 0.28ab	13.38 ± 0.44a	11.88 ± 0.28b
EDL (mg)	8.00 ± 0.40	7.75 ± 0.13	7.88 ± 0.24	8.18 ± 0.24	7.28 ± 0.18
TA (mg)	36.03 ± 1.20a	35.27 ± 1.23ab	35.76 ± 0.80ab	36.31 ± 0.86a	31.99 ± 0.83b
Fat (mg)	313.60 ± 32.41a	238.80 ± 43.78ab	270.10 ± 23.88ab	340.00 ± 35.25a	175.40 ± 30
Heart (mg)	102.9 ± 3.32	100.80 ± 1.80	102.40 ± 4.8	105.30 ± 2.98	105.20 ± 3.80
Liver (mg)	729.30 ± 31.08a	804.30 ± 31.68ab	825.40 ± 45.73ab	896.50 ± 45.73bc	1043.00 ± 47.29c
**Spleen (mg)**	78.93 ± 4.86a	67.14 ± 3.75a	95.86 ± 7.99a	118.1 ± 5.81a	328.20 ± 28.32b

### Global gene expression analysis showed substantial transcriptome shift in the high-tumor group only

**Fig. 1:**
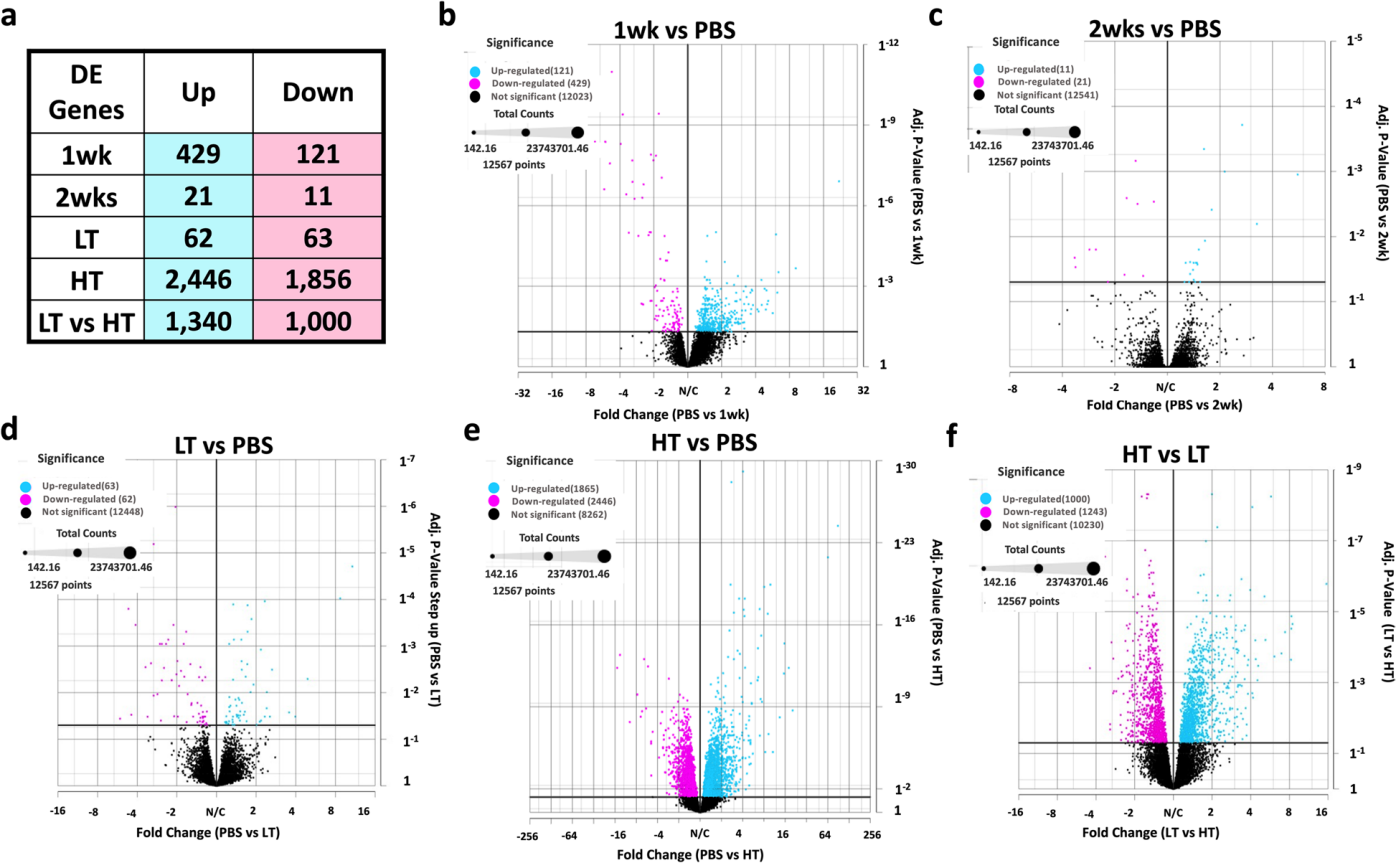
**a** Total number of Differentially Expressed Genes (DEGs) compared to PBS control. LT vs HT comparison was added. Volcano Plots for 1 week (1wk) (**b**), 2 weeks (2wk) (**c**), Low Tumor (LT) (**d**), and High Tumor (HT) (**e**), compared to PBS control. HT compared to LT (**F**) was added. Adjusted *P*-value < 0.05

**Fig. 2:**
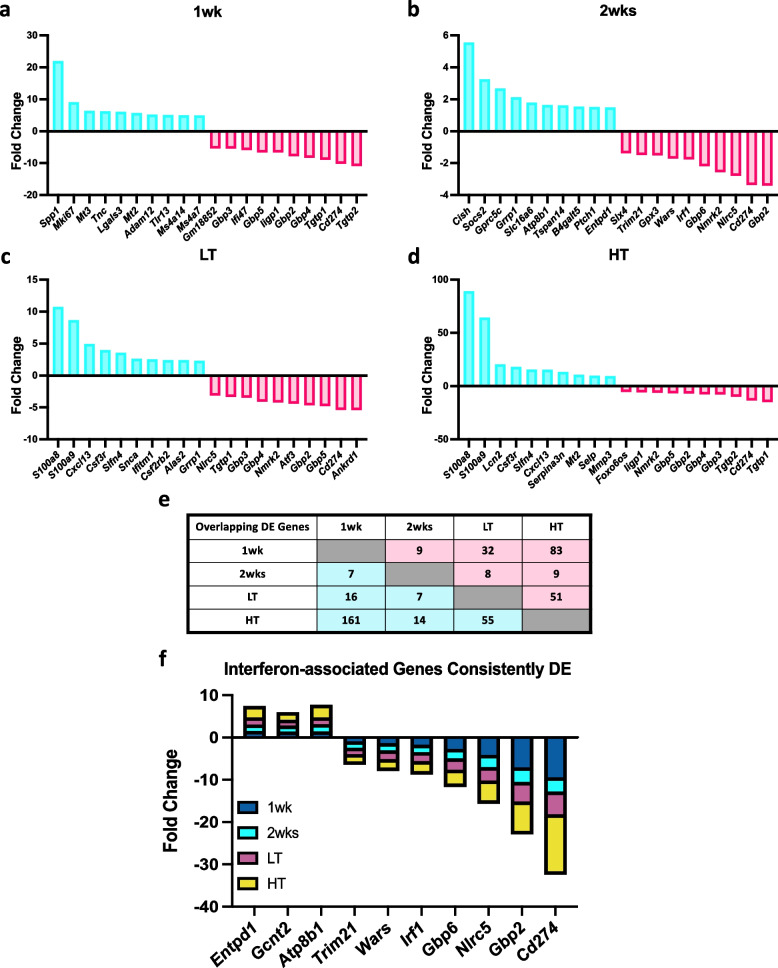
Top 10 Up- and Downregulated DEGs. Tumor-bearing 1 week (1wk) (**a**), 2 weeks (**b**) Low Tumor (LT) (**c**), and High Tumor (HT) (**d**) compared to PBS control. A total number of matches of up and down-regulated Differentially Expressed (DE) Genes compared to PBS control across all comparisons, LT vs HT comparison was added (**e**). Consistently DE genes across all cancer time points are associated with interferons (**f**). Adjusted *P*-value < 0.05

We conducted a global gene expression analysis using high-throughput RNA sequencing data from the gastrocnemius muscle of female mice at 1wk, 2wk, LT, and HT time points following tumor allograft (8 animals/condition). The choice of the gastrocnemius muscle was intentional, as it is a heterogeneous fiber type muscle and enables us to make direct comparisons with our previous male transcriptomic study in gastrocnemius muscles of LLC-bearing mice [12]. This ensures that our findings are consistent with existing research and allows for a comprehensive understanding of gene expression in the context of CC. To identify muscle transcriptome shifts in CC, we performed RNA-seq of gastrocnemius muscle at all time points. We identified 2,958 upregulated and 2,052 downregulated DEGs across all time points (Fig. 1a, adj. *P*-Value < 0.05). Most DEGs were in the HT group (83% of total DEGs) (Fig. 1b-e adj. *P*-Value < 0.05). DESeq2 analysis showed 2,446 upregulated, and 1,856 downregulated DEGs in HT compared to PBS while all the other time points combined showed 512 up- and 195 downregulated DEGs (Fig. 1b-e, adj. *P*-Value < 0.05). Comparing LT against HT we noted 1,000-up and 1,343 downregulated DEGs (Fig. 1f, adj. *P*-Value < 0.05). The top ten up- and downregulated genes of each timepoint are in Fig. 2a-d. The top ten upregulated genes showed an elevation in genes associated with an antioxidant action (*Mt3, Mt2*) along with cell structure-associated genes (*Lgals3, Adam12*) in 1wk mice. Interestingly, various Guanylate Binding Proteins (*Gbp2*, *Gbp3, Gbp4 Gbp5*) were downregulated in 1wk mice and remained downregulated throughout later time points along with other immunity and interferon-associated genes (*Entpd1, Gcnt2, Atp8b1, Trim21 Wars, Irf1, Nlrc5, Cd274)* (Fig. 2a-d).

To investigate differences and similarities in DEGs across time points, we investigated distinctions in DEGs across time points (Fig. 2e, f). Overlapping DEGs between tumor-bearing comparisons are found in Fig. 2e. We subsequently identified 10 overlapping DEGs (3 upregulated and 7 downregulated) in all tumor-bearing groups compared to the control (Fig. 2f). Overlapping genes were associated with immune response, specifically Type-II-interferon signaling.

### Pathway analysis revealed biphasic biological alterations in CC development

**Fig. 3:**
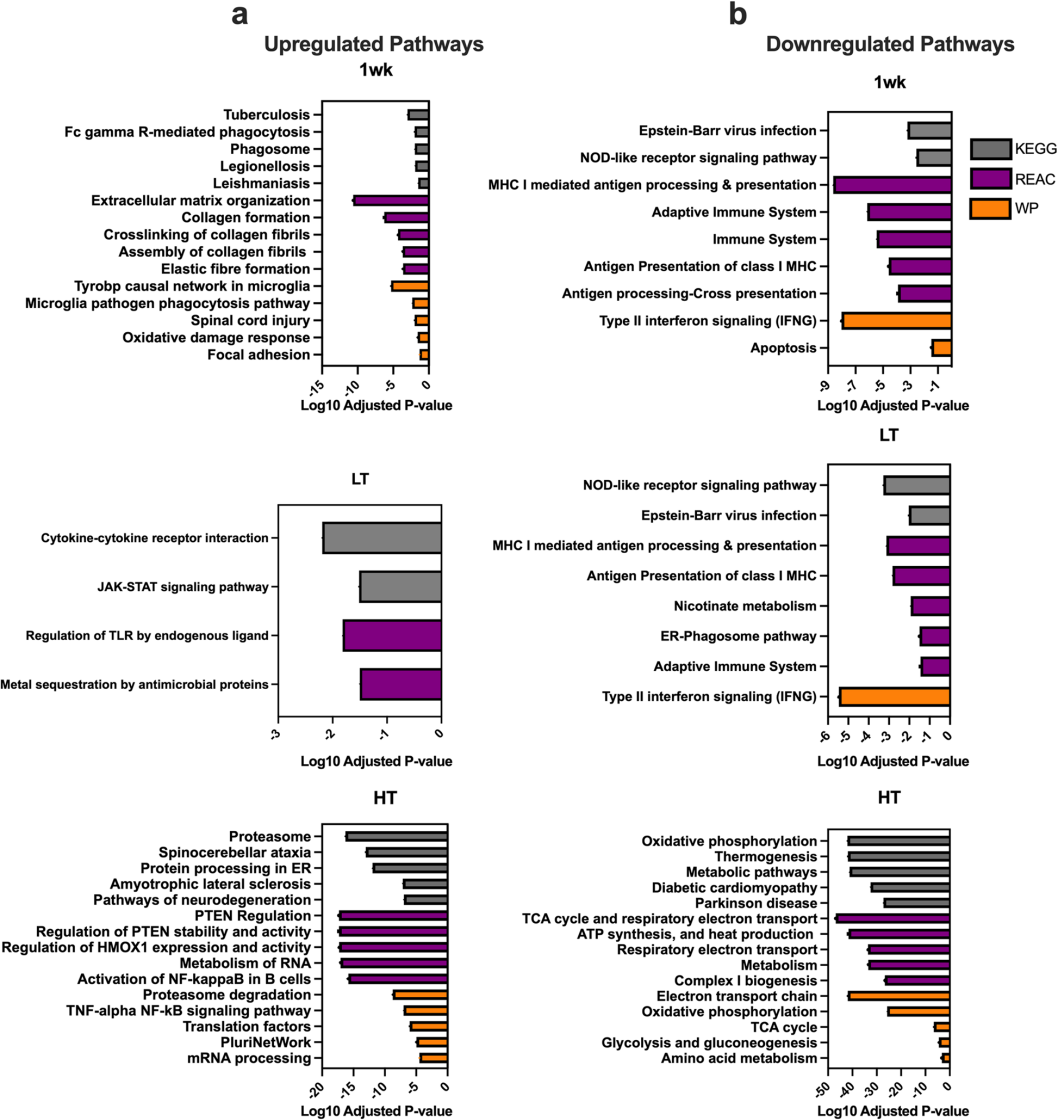
Top 5 Up- (**a**) and Downregulated Pathways (**b**) for KEGG Reactome and WikiPathways. Tumor-bearing 1 week (1wk), Low Tumor (LT), and High Tumor (HT) compared to PBS control. Adjusted *P*-value < 0.05

Next, we explored the functional ontology of the altered gene networks. The top up- and downregulated pathways (up to five) of KEGG, Reactome, and WikiPathways biomolecular pathways libraries are shown in Fig. 3a, b. Phagosome-associated pathways were among the upregulated pathways in the 1wk group, along with multiple pathways suggesting ECM remodeling, changes in cell structure, and oxidative stress response. At two weeks after tumor introduction, the JAK-STAT pathway was the only significantly upregulated pathway (not shown), which remained significant in the LT group, along with the cytokine-cytokine receptor interaction pathway. In the HT group, we identified upregulation of Proteasome, mRNA processing, Translation processing, and multiple inflammation-associated pathways. Multiple immune system-related pathways were downregulated at 1wk along with type-II-interferon pathways also consistently dysregulated in LT and HT groups. The most downregulated pathways in HT were associated with metabolism and mitochondrial systems, including oxidative phosphorylation (OXPHOS), ATP synthesis, TCA cycle, and Electron transport chain (ETC) pathways. When compared to analysis utilizing a background gene list, the top pathways and biphasic characteristics were similar, confirming our results (Supplementary File 3).

### A comparison of RNA sequencing to MitoCarta shows striking alterations to mitochondria-associated genes in CC

**Fig. 4:**
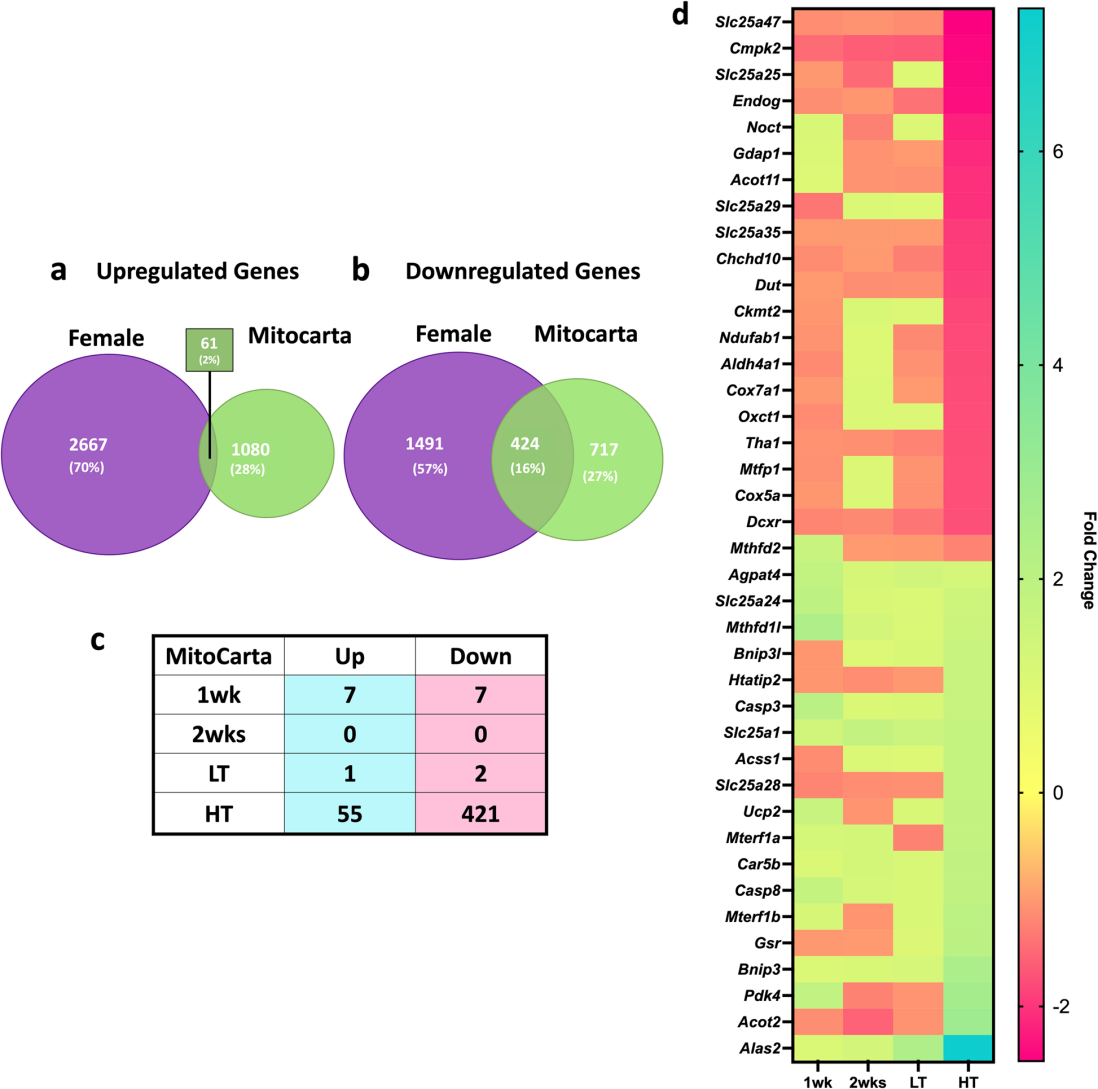
DEGs Tumor-bearing Female and MitoCarta 3.0 Cross-reference. Venn Diagram of Upregulated Genes (**a**), Venn Diagram of Downregulated Genes (**b**), Matching Genes (**c**) of Female and MitoCarta 3.0 datasets, Top 20 Up- and Downregulated matching Genes of Tumor-bearing Female and MitoCarta 3.0 (**d**) genes organized from lower to higher FC in HT

**Fig. 5:**
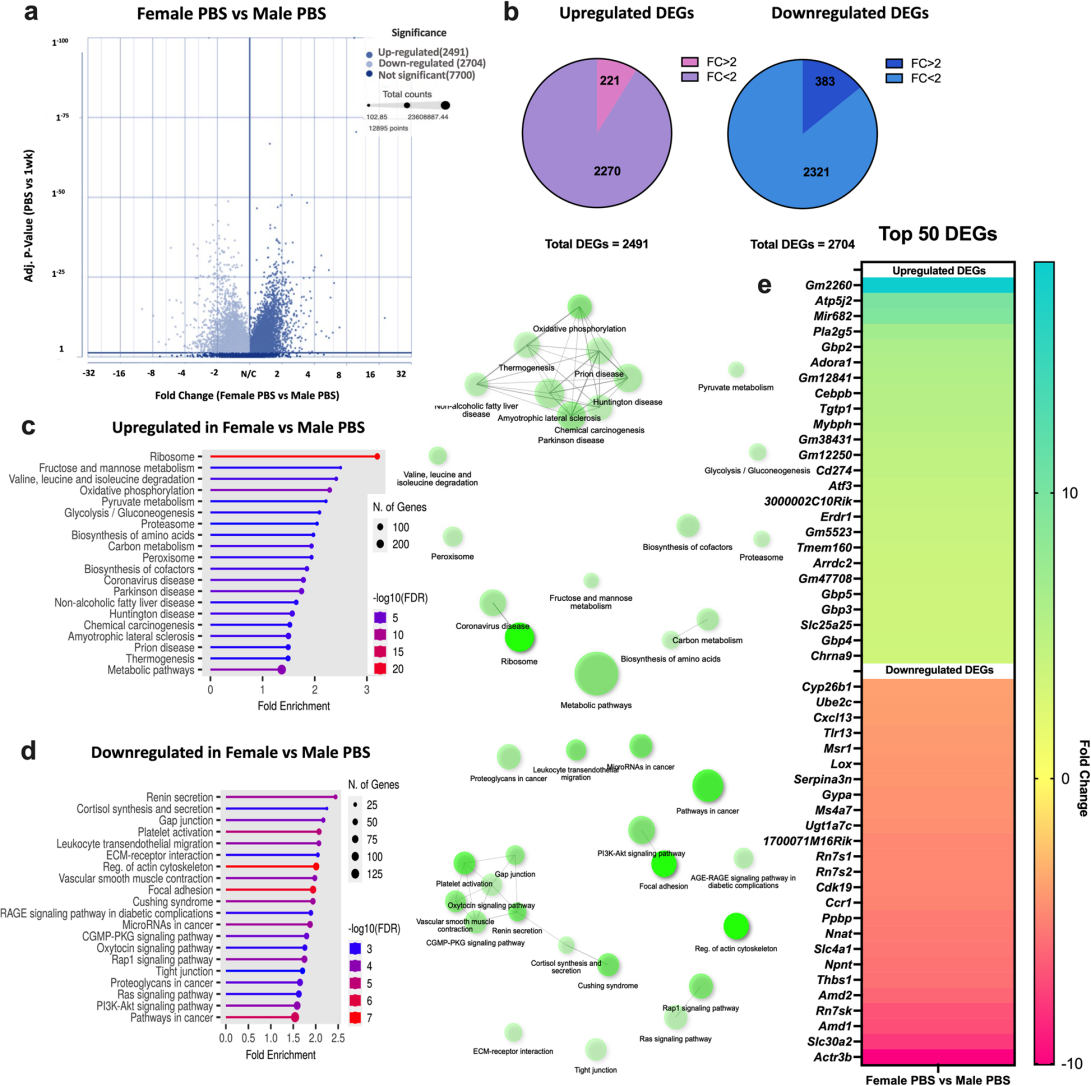
Female and Male Basal Comparison. Volcano plot of DEGs (**a**), Total DEGs and FC > 2 cutoff (**b**), Top 20 Upregulated Pathways and Pathway Network (ShinyGO, **c**) Top 20 Downregulated Pathways and Pathway Network ( ShinyGO, **d**), Top 50 DEGs (**e**). Adjusted *P*-value < 0.05

Considering the substantial disruption of mitochondria-associated pathways, we compared the current dataset with MitoCarta 3.0, composed of 1,141 known mitochondrial-associated genes [28]. Altogether, overlapping DEGs represent 6% and 41% of MitoCarta genes that were up- and downregulated with LLC, respectively (Fig. 4a, b). Very few genes matched the MitoCarta in the pre-cachectic groups. In the HT group, 55 up- and 421 downregulated genes were matched (Fig. 4c). The top 20 MitoCarta matched are shown in Fig. 5d including *Alas2, Bnip3,* and *Casp8* (Fig. 4d). *Cox5a,* a subunit of Cytochrome C Oxidase and multiple mitochondrial transporters genes were among the top 20 downregulated genes, including *Slc25a47, Slc25a25,* and other *Slc* genes (Fig. 4d).

### Comparison of RNA sequencing to males revealed biological sex dimorphism in transcriptomic alteration in CC

**Fig. 6:**
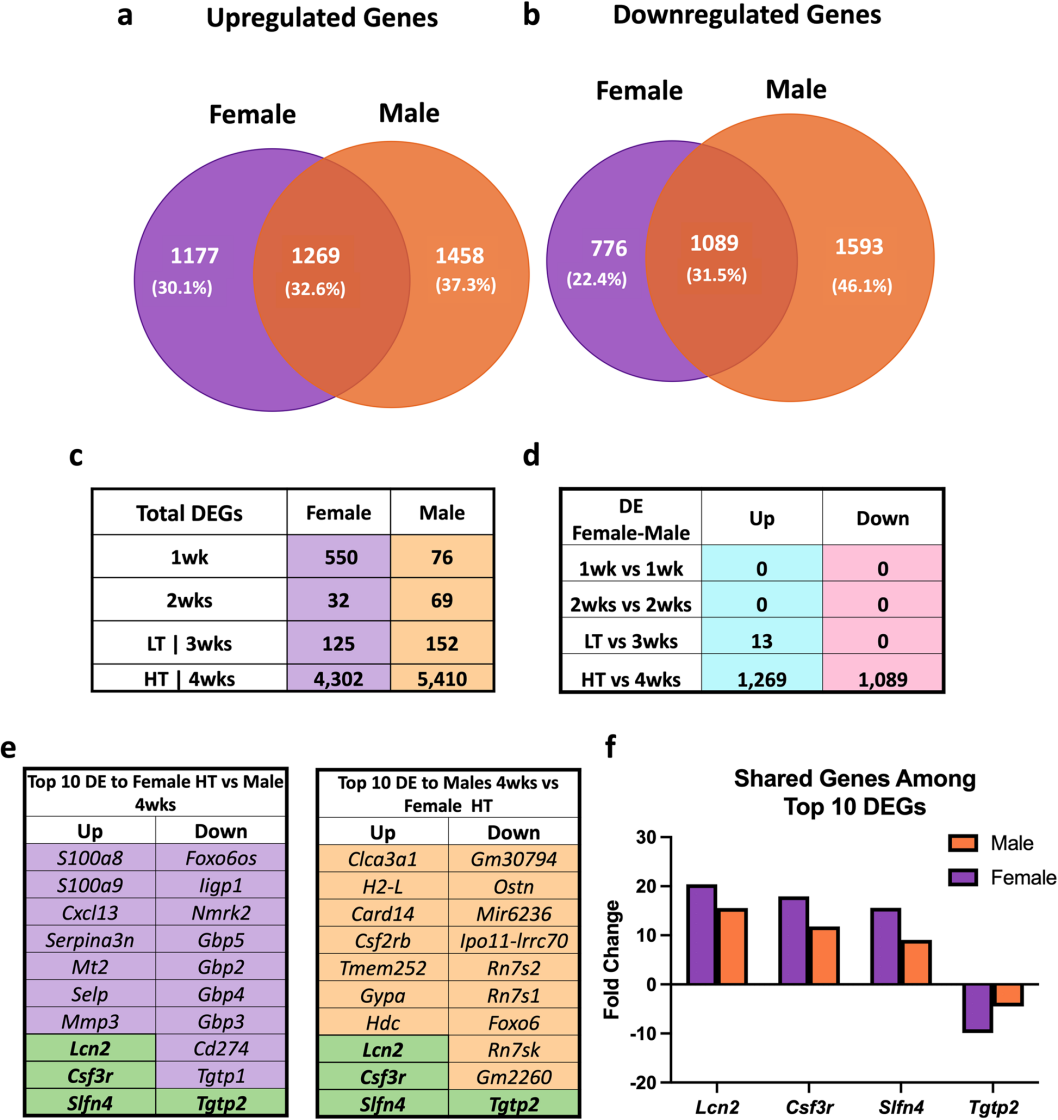
Female and Male Comparison. Venn Diagram of Upregulated Genes (**a**), Downregulated Genes (**b**), Total DEGs at each time point for females and males (**c**), Matching DEGs (**d**) of Female and Male datasets, Top 10 Genes Female HT vs 4wks (**e**), Top 10 Genes Male 4wks vs Female HT. Shared DEGs FC (**f**). Purple cells = genes unique to females, orange cells = genes unique to males, and green cells = common genes between biological sexes. Adjusted *P*-value < 0.05

As biological sex plays an important role in CC [10, 17, 18], we cross-referenced female data with our previously published study utilizing LLC-induced cachexia in males [12]. We previously reported phenotypic data for this male subset including an ~ 17% lower gastrocnemius mass in cachectic animals (four weeks of tumor development) compared to PBS control [12, 19]. In males, non-skeletal muscle tissues that are sensitive to cachexia development showed similar changes. Specifically, we observed splenomegaly (350% greater compared to the control group), hepatomegaly (35% greater than the control), and fat loss (34.5% lower compared to the control group) after three to four weeks of tumor development [19, 29]. For consistency, we reanalyzed male datasets using matching parameters as in the current study. To gain insight into intrinsic transcriptional differences in skeletal muscle across biological sexes in the absence of tumor-bearing conditions, we compared female PBS to male PBS (Fig. 5). Our analysis identified a total of 2491 upregulated and 2704 downregulated DEGs in females compared to males PBS (adjusted *P*-value < 0.05 and fold change (FC) > 1) (Fig. 5a). To further investigate the extent of these differences, we applied a more stringent FC cutoff of > 2, which revealed 221 upregulated and 383 downregulated DEGs (Fig. 5b). To understand the biological implications of these gene expression alterations, we performed a pathway analysis. This revealed that in female PBS, there was an upregulation of pathways related to *Ribosomes*, several *Metabolic processes*, and *Proteasome*-associated pathways. Conversely, there was a downregulation of pathways related to *cortisol synthesis and secretion, gap junction, ECM-receptor interaction, regulation of actin cytoskeleton, focal adhesion, and PI3K-Akt signaling* pathways (Fig. 5c, d). The top 50 up- and down-regulated genes are illustrated in Fig. 5e. In tumor-bearing groups, there was a 31% overlap of DEGs, (genes displayed in several time points were accounted only once for percentage calculation), with a total of 1282 up- and 1098 downregulated overlapping DEGs, respectively (adj. *P*-Value < 0.05, Fig. 6a, b). The total DEGs for females and males indicate an early transcriptional response to tumor presence in females with more DEGs in the presence of global cachexia, while males lack the early response and show higher DEGs at the later stage (Fig. 6c). No overlapping genes were found in 1- and 2-wks groups in females and males (Fig. 6d). Only 13 matching upregulated genes were identified between female LT and male 3wks groups, while in groups that displayed cachectic phenotype (female-HT and 4-wks-male), there were 1269 up- and 1089 downregulated genes matching between female and male LLC-bearing mice (Fig. 6d). Amongst the top ten shared up- and downregulated genes for females and males, there were three (*Lcn2, Csf3r, Slfn4)* upregulated and one (*Tgtp2)* downregulated common gene (Fig. 6e, f). Downregulation of *Gbp* genes was exclusive to female LLC-bearing mice (Fig. 6e, f).

**Fig. 7:**
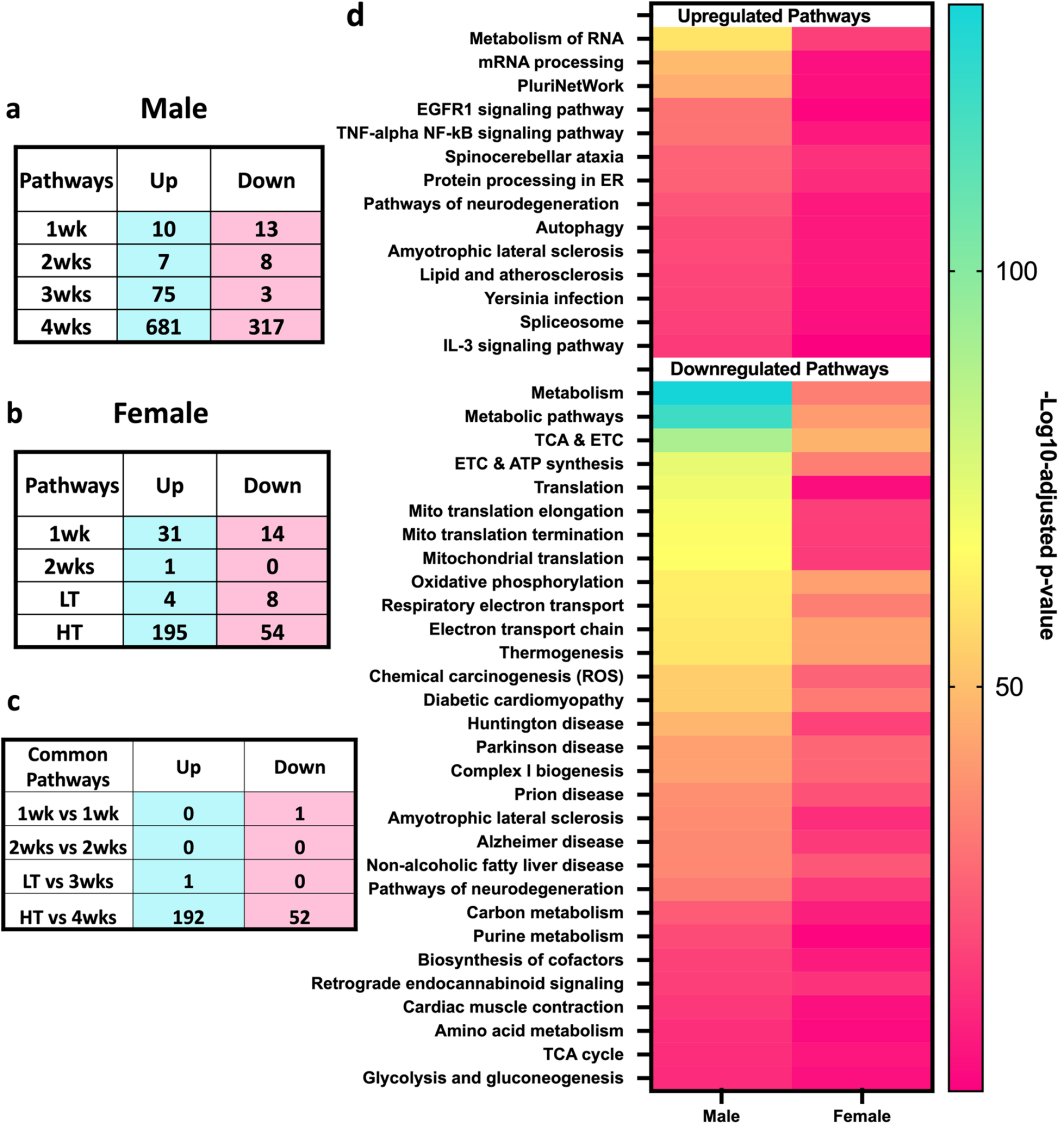
Female and Male Pathway Comparison. Numer of dysregulated pathways in males (**a**), Number of dysregulated pathways in females (**b**), Matching Pathways in males and females (**c**) Top shared pathways in males and females (**d**)

To understand the differences and similarities of CC-induced transcriptomic shifts in females and males, we compared dysregulated pathways (Fig. 7). Both G:Profiler and ShinyGO 0.77 analysis resulted in similar outcomes. We found only one common downregulated pathway in 1wk groups and one common upregulated pathway in female LT and 3wks male groups. Most overlapping pathways were in the cachectic groups (female HT and male 4wks), with a total of 192 and 52 up-and downregulated respectively (Fig. 7c). Considering the cachectic groups in both males (4 weeks) and females (HT) displayed the larger transcriptomic alterations, we focused subsequent analyses on 4 weeks and HT groups only. We next analyzed the top 20 disrupted pathways of each KEGG, Reactome, and WikiPathways, observing 37.5% of pathways were shared by cachectic females and males (Fig. 7d, one repeated pathway was excluded). Amongst shared pathways, we identified upregulation of pathways associated with inflammation, metabolism of RNA, mRNA processing, autophagy, and exercise-induced circadian regulation, accompanied by downregulation of pathways associated with metabolism and mitochondrial systems (Fig. 7d). We noted a more potent disruption of shared pathways in male compared to female mice, with male 4 weeks showing a more robust significance of the dysregulated pathways (Fig. 7d, Adj. *p*-value < 0.05). Unique dysregulated pathways for females and males are in Additional file 1a-b and show female-unique upregulation of oxidative stress response, auto-degradation of the E3 ubiquitin ligase COP1, and proteasome among others. Upregulation of insulin signaling, cellular responses to stress, and metabolism of proteins among others, were unique to males (Additional File 1). Additionally, distinct upregulation of *Interferon Pathways* including 15 genes (*Ube2l6, Isg15, Flnb, Ptpn6, Eif4g1, Eif4a3, Plcg1, Pias1, Abce1, Ptpn1, Kpna1, Ppm1b, Arih1, Kpnb1, and Ptpn11*) was noted in 4wks male (Data not shown, *p* = 0.0001). Unique downregulated pathways to females displayed a relationship with mitochondrial pathways (Additional file 1a), while male-only downregulated pathways included DNA replication and repair, estrogen signaling, and ribosomal pathways, in which 40% of genes in this pathway were dysregulated, including many Mitochondrial Ribosomal Proteins (MRBLs) and Ribosomal Proteins (RPSs) genes (Additional file 1b).

**Fig. 8:**
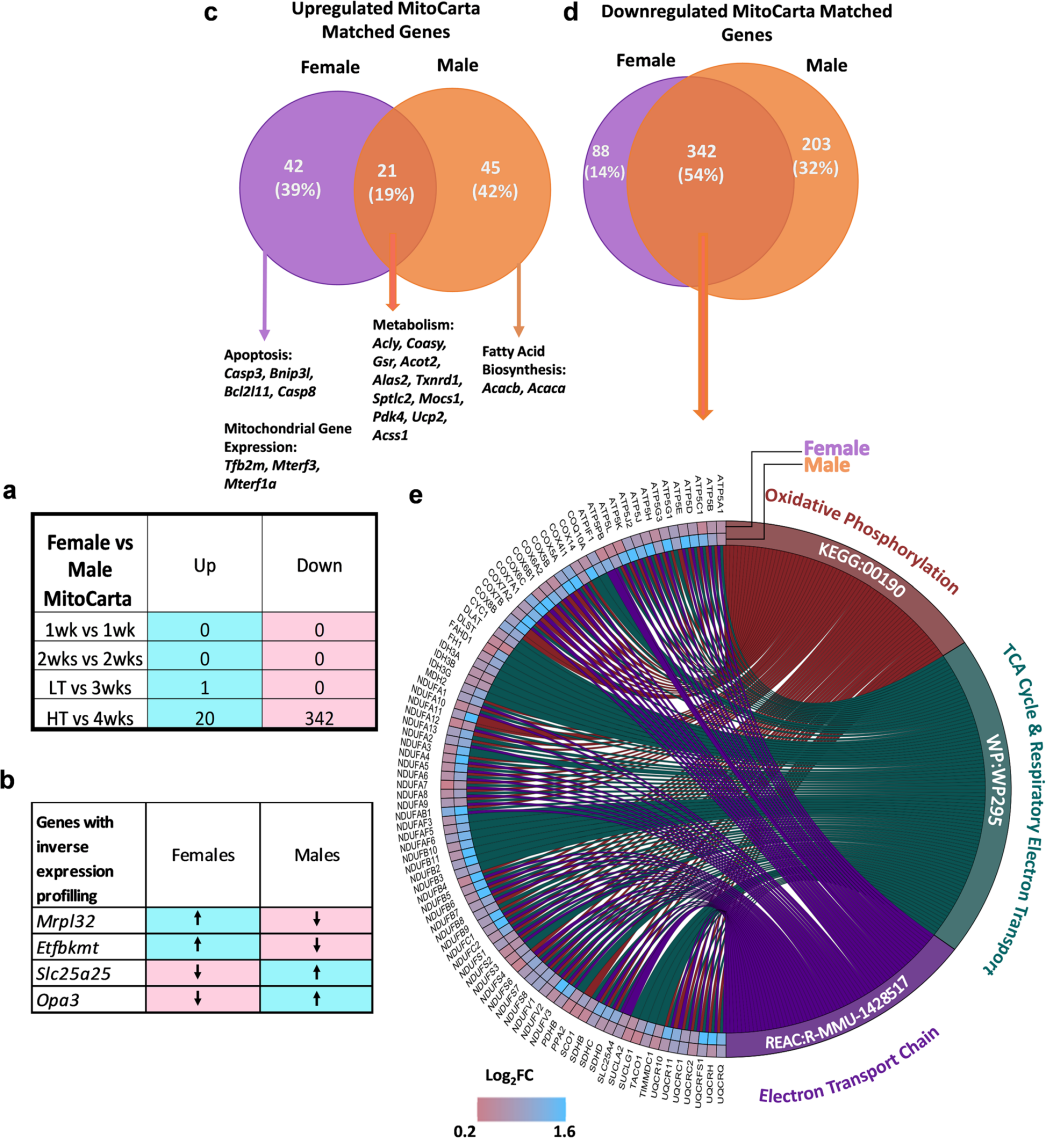
Biological dimorphism with a focus on mitochondrial genes. Total number of shared mitochondrial genes in females and males at all time points (**a**). Inversed expression of four mitochondrial genes between biological sexes across all time points (**b**). Venn diagram of up-and down-regulated mitochondrial genes in females High tumor (HT) and males 4wks (**c** & **d**). Chord diagram display differences in Log_2_FC in common downregulated mitochondrial genes (**e**)

After the observation of many mitochondrial-associated pathways being a dominant factor in the comparison of male and female mice undergoing CC, we inquired whether there were differences in MitoCarta matched genes in CC between biological sexes (Fig. 8). Comparably with global gene expression, the most commonalities were found in the HT vs 4wks comparison, with 20 up- and 342 downregulated shared mitochondrial-associated genes (Fig. 8a). Four genes displayed inverse expression profiling between biological sexes (Fig. 8b). Figure 8c shows ~ 19% share of total upregulated mitochondrial genes of females and males, highlighting the unique upregulation of apoptotic genes and mitochondrial gene expression-related genes in females, while fatty acid biosynthesis-related genes were uniquely upregulated in males. Downregulated mitochondrial genes displayed 54% shared genes between sexes (Fig. 8d). Figure 8e shows a distinction in Log_2_FC between the mitochondrial genes associated with the three top common pathways between biological sexes, highlighting a more prominent impact in males (higher Log_2_FC) compared to females.

## Discussion

Effective treatments for CC remain lacking. Unfortunately, most preclinical studies have been historically performed in males, overlooking likely biological sex dimorphisms. Our group and others have shown differences in CC development between biological sexes [10, 17–21], demonstrating the necessity to study underlying mechanisms of CC unique to each biological sex. Here, we first evaluated the time course of transcriptomic alterations in response to LLC-induced CC in female mice. We then directly compared cachexia-induced transcriptomic alterations between the current dataset in female LLC-bearing mice with prior data from our laboratory in male LLC-bearing mice [12]. In females, we observed muscle transcriptomic shifts one week following tumor allograft. Most importantly, major transcriptomic alterations occurred with the onset of the whole-body cachectic phenotype despite the relative preservation of gastrocnemius muscle mass in females. We further show the disruption of important pathways involved in detriments to muscle function and health with cachectic development, including *cell structure, autoimmune system, protein ubiquitin, JAK-STAT pathways, and oxidative metabolism* in female mice. Cross-referencing female and male data [12] demonstrate only a ~ 32% overlap in DEGs of cachectic mice between biological sexes. Our data demonstrate unique downregulation of type-II-interferon genes amongst the top DEGs and pathways to female mice. Overall, data suggest a specific biological sex signature to CC progression.

### Overview of transcriptomic shifts in female mice undergoing LLC-induced cachexia

Our experimental model was successful in demonstrating key components of cachectic phenotype, including loss of fat mass, TA and soleus muscle wasting, and splenomegaly. Transcriptional profiling of gastrocnemius muscle showed strong transcriptomic alteration one week after cancer inoculation. Moreover, ~ 86% of all DEGs were observed only after global cachectic phenotype occurred. These findings are consistent with prior reports, confirming large transcriptomic shifts concurrent with the onset of the cachectic phenotype [10, 12, 30]. Similarly, prior research showed transcriptomic alterations in early and late stages of C26 colorectal and pancreatic ductal adenocarcinoma cancer-induced cachexia, despite mitigated muscle mass loss at early cachexia stages in females [10, 30]. Altogether, a large degree of transcriptomic shifts occurs with the onset of cachexia.

### Consistent dysregulation of GBPs across the development of CC in females

We next cross-referenced DEGs between all tumor-bearing groups revealing the 1wk and HT groups shared the most similarities. Interestingly, we observed only ten genes consistently dysregulated across all time points. These ten genes were associated with downregulation of Type II interferon signaling, including upregulation of three type II interferon repressors and the downregulation of seven genes associated with type II interferon activation. Furthermore, several Gbp genes were downregulated across all time points. Specifically, Gbps-2, -3, -4, -5, and -6 were amongst the ten most down-regulated genes across tumor-bearing groups. Gbps are a GTPase family strongly induced by interferons and can contribute to cell survival by inhibiting apoptosis [31]. Previous studies have shown Type II interferon signaling is downregulated in the muscle of aged mice undergoing impaired regeneration and inefficient muscle regeneration in interferon null mice, suggesting a role in muscle mass regulation [32, 33]. Moreover, a recent study utilizing single-cell RNA-seq revealed upregulation of interferon-induced GBP through endothelial cells within the skeletal muscle is a key mechanism of aging-associated muscle loss (sarcopenia) [34]. The role of Type II interferon signaling in muscle mass regulation is not yet fully understood, but this dataset supports a potential role for Type II interferon, specifically downregulation of *Gbps,* in the female-specific protection against CC muscle wasting.

### Pathway analysis: biphasic transcriptomic alterations in females

Consistent with prior work in pancreatic cancer patients [35], functional ontology revealed upregulation of pathways related to extracellular matrix (ECM) remodeling and cell structure in the 1wk group, including genes involved in collagen biosynthesis, deposition, and focal adhesion, such as several Col-family genes. In our recent publication [36], we have expanded upon our findings of enhanced collagen deposition in LLC-bearing mice, including data from a larger cohort of both male and female animals. Dysregulation in cell structure and induction of ECM remodeling, including collagen deposition and fibrosis, has been linked to poorer prognosis for cachectic patients [35, 37] and are observed in other tissues (i.e., cardiac muscle) [38], representing a hallmark of atrophy [15]. Notably, increased collagen deposition at the skeletal muscle endomysium is unique to cachectic cancer patients, whereas non-cachectic cancer patients do not exhibit collagen deposition [39]. Minimally disrupted pathways were noted in middle time points (2 wk and LT) other than the JAK-STAT pathway, a known mediator of cachectic wasting [30, 40] associated with the acute phase response commonly observed in cachexia [30, 40].

Imbalanced protein turnover is a hallmark of muscle wasting [41]. Not surprisingly, *proteasome* was the most upregulated pathway in the HT group. The timing of the proteasomal system induction agrees with our prior findings [17] and ties the induction of major catabolic signaling cascades with the onset of the cachectic phenotype. Similarly, the downregulation of multiple *immune system-associated* genes and pathways was observed one week following tumor allograft persisting through tumor development. Interestingly, inflammation and immune system factors such as Nuclear Factor kB (NFkB) and Type II interferon are inversely regulated by ubiquitin–proteasome systems [42], likely explaining concomitant downregulation of the immune system with increased *proteasome* activity. Our current findings regarding the induction of the proteasome and dysregulation of immune-related functions are consistent with our prior report in males [12, 18]. This suggests that there may be a delay in the onset of muscle wasting in females with a similar global cachectic phenotype compared to their male counterparts, who experienced muscle loss [12]. These results provide valuable insights into the mechanisms underlying muscle wasting and highlight potential sex-based differences that could inform the development of targeted interventions to manage this condition.

### Over 40% of Mitochondrial Genes are disrupted in cachectic females

We previously documented impaired mitochondrial health in multiple atrophy models, including LLC-induced cachexia [17, 19, 22, 43]. Specifically, mitochondrial degeneration preceded atrophy in male mice undergoing either LLC-induced cachexia or disuse atrophy, while females largely protect mitochondrial health until the onset of muscle atrophy [17, 19]. Herein, we noted strong dysregulation in mitochondrial pathways including *oxidative phosphorylation, metabolic pathways, and electron transport chain*, among others with the onset of cachexia itself. Cross-referencing DEGs with the MitoCarta 3.0 shows alterations in 41% of MitoCarta genes suggesting large disruptions in mitochondrial gene expression correlate with the onset of mitochondrial dysfunction and cachexia [17]. In males, we have reported early transcriptional alterations matching mitochondrial functional impairment preceding skeletal muscle loss [12, 19]. While mechanisms behind biological sex differences in cachexia remain largely elusive, the ability of female mice to protect muscle mitochondria during the early stages of development may provide one possible explanation.

### Only one-third of DEGs are shared between biological sexes

Most strikingly when comparing DEGs between cachectic male and female mice ~ 2/3 of all DEGs were biologically sex specific. While acknowledging the limitations of not observing atrophic changes in the gastrocnemius muscle of female mice, its selection for our study enables us to make more accurate biological sex comparisons with previously published data [12]. Furthermore, recent studies have indicated that female mice may exhibit a delayed or protective response against CC when compared to male mice [10, 17, 19]. Thus, we hypothesize that our study design, which focuses on the non-cachectic gastrocnemius muscle in the context of global cachexia, provides an opportunity to investigate the transcriptional mechanisms that contribute to the delayed response to tumor burden in female mice.

Furthermore, this observation aligns with recent work in the KPC model of cachexia where in “late-stage” (i.e., cachectic) where a similar portion of DEGs was shared between biological sexes [10]. Notably, the genes *Lcn2, Csf3r*, and *Slfn4* are among the most upregulated genes shared by both males and females undergoing CC. Increased serum levels of *Lcn2,* as well as elevated mRNA levels in skeletal muscle, have been linked to the MDX mouse model of Duchenne Muscular Dystrophy [44]. Moreover, Lcn2 is associated with impaired skeletal muscle regeneration [45], Csf3r with inhibition of cellular proliferation, and Slfn4 with cell cycle arrest [46]. These findings suggest common mechanisms related to impaired cellular proliferation, regeneration, and cell cycle arrest, may trigger CC-induced muscle impairments across biological sexes.

Similarly, across functional ontology only 37% of male pathways matched with females demonstrating a larger transcriptomic disturbance in males. Among shared pathways, many were largely expected and associated with protein turnover and energy metabolism. Considering unique pathways, downregulation of multiple pathways associated with *DNA damage repair* and *ribosomal pathways* (including both mitochondrial ribosomal protein and ribosomal protein-encoding genes) appeared specific to males, suggesting DNA maintenance and ribosomal activity play an important role in the protection in females but not male mice undergoing cachexia. This data is suggestive of the role of ribosomal activity, enriched in healthy females compared to males, in muscle mass regulation in cachexia discussed in previous studies showing reduced ribosomal capacity is associated with the loss of muscle mass [47]. Females did not display dysregulation of ribosomal pathways, accompanied by preservation in gastrocnemius mass, while the opposite was noted in males, suggestive of a protective role of muscle mass in the maintenance of ribosomal function.

**Fig. 9:**
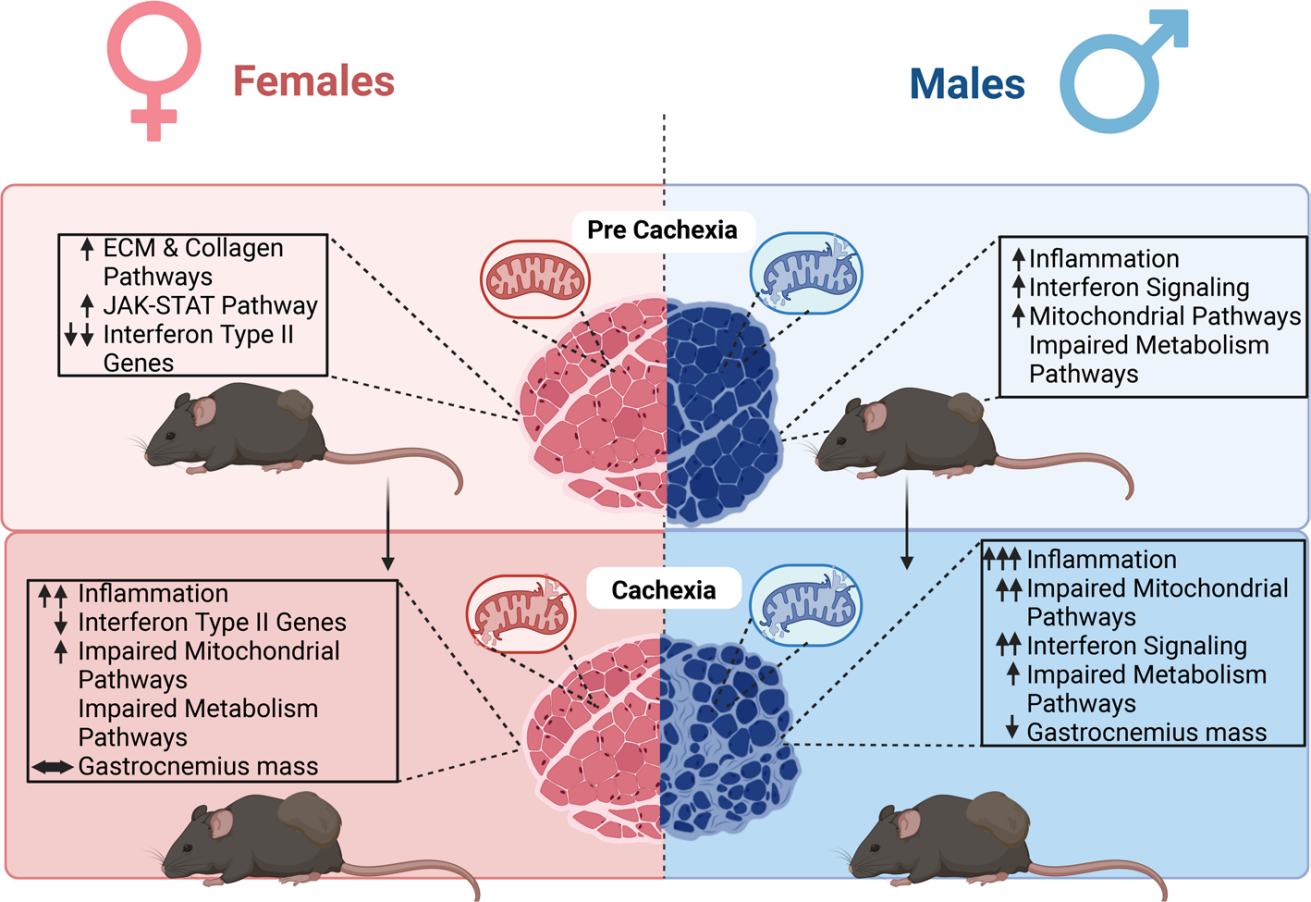
Summary figure. Females and males display different timelines in the transcriptional response to tumor development and cancer cachexia. Females display an early dysregulation in extracellular matrix remodeling and cellular structure-associated pathways followed by an increase in JAK-STAT pathways-associated gene expression. Interestingly, besides the maintenance of gastrocnemius muscle mass, downregulation of interferon type II genes is unique to females through cancer cachexia development. Delayed transcriptomic changes in mitochondrial pathways match functional mitochondrial impairments previously reported. Males show upregulation inflammation pathways, and interferon signaling genes at pre-cachectic and cachectic states. Additionally, males show early disruption in mitochondrial function that precedes the loss of gastrocnemius mass and major transcriptomic changes in mitochondrial genes. Invert expression profiling of interferon-associated genes between biological sexes represents a transcriptomic signature to each biological sex in response to cancer cachexia development and suggests a potential target for muscle loss during cancer-induced cachexia. Created with BioRender.com

Additionally, while female mice show consistent downregulation of interferon type II associated genes in multiple time points of CC development, males show upregulation of interferon genes at 3- and 4-weeks following tumor allograft, raising the possibility of a novel interferon role in cancer-induced atrophy in male mice. Meanwhile, females displayed unique upregulation of *exercise-induced circadian regulation,* including *Pura,* and *Ncoa4*, which are associated with the regulation of DNA replication, preventing inappropriate DNA synthesis and replication stress [48]. Even though the roles of *Pura* and *NCoa4* in skeletal muscle circadian biology are not fully elucidated, their roles in the regulation of circadian events in neural systems [49], and autophagic degradation *Arntl* (aka. *Bmal1*) [50] were recently unveiled. Moreso, females showed upregulation of *Oxidative Stress Response,* including important antioxidant-associated genes including *Sod1* and *Cat* [51] which may partially explain the relative preservation of mitochondrial health noted in females [17] compared to males undergoing CC. Altogether, data suggest key biological sex dimorphisms in functional and intrinsic skeletal muscle alteration and therefore sex-specific mechanisms of cachexia (Fig. 9).

## Conclusion

Despite the potential importance of biological differences between males and females in health and disease, most pre-clinical research on CC is still conducted in male specimens. This study aimed to investigate female transcriptomic alterations in response to tumor presence and to compare them to those of male mice. Additionally, our findings contribute to a growing body of evidence supporting biological sex differences in the development of CC. Here, about two-thirds of all DEGs were biologically sex-specific. We also observed downregulation of interferon-associated genes which was unique to females and coincides with the preservation of skeletal muscle mass in the presence of systemic cachectic phenotype. In contrast, males showed elevated interferon signaling, along with marked impairments in energy metabolism pathways and muscle mass loss. Although this study is limited to preclinical analysis and the use of a single cancer type, our findings represent a potential new therapeutic target to mitigate poor CC outcomes by reducing interferon signaling in skeletal muscle environments. Overall, our data strongly suggest sex-specific mechanisms of CC which must be considered in understanding this debilitating condition and developing appropriate and effective therapeutic approaches.

## Materials and methods

### Animal interventions

Female C57BL6/J mice (Jackson Laboratories, Bar Harbor, ME; *n* = 40, 8/condition) from a larger study were randomly selected [17]. Phenotypic statistics for this subset were performed to validate the representation of the larger dataset. Animals were kept on a 12:12-h light–dark cycle and given access to normal rodent chow and water for the duration of the study. All animal protocols were approved by the University of Arkansas Institutional Animal Care and Use Committee, in accordance with the ethical standards (1964 Declaration of Helsinki).

#### Lewis lung carcinoma allograft

Lewis lung carcinoma (LLC) cells (ATCC, CRL‐1642) were grown as described [17]. At eight weeks of age, female mice were subcutaneously injected with either LLC cells (1 × 10^6^) suspended in 100μL PBS or equal volume sterile PBS (control) to the right hind flank. Tumors were allowed to develop for 1, 2, 3, or 4 weeks; 4 weeks is a time point commonly associated with mild cachexia in this model, majorly utilizing male subjects [18, 19]. As we noticed a dichotomous pattern in tumor weight, 3 and 4wk mice were regrouped into low (LT, tumor-weight ≤ 1.2 g) and high (HT, tumor-weight ≥ 2 g) tumor-bearing [17] *n* = 8/group. Five animals were moved from 4 weeks to the LT group, and six animals were moved from 3 weeks to the HT group. Control (PBS) mice were age-matched with 12-week-old mice.

### Tissue collection

Mice were anesthetized with isoflurane before euthanasia and tissue wet weight of gastrocnemius, plantaris, soleus, extensor digitorum longus (EDL), tibialis anterior muscles of both limbs, along with heart, spleen, liver, and gonadal fat were assessed. Samples were snap-frozen in liquid nitrogen and stored at -80 °C for further utilization.

### RNA isolation and quality check

Total RNA was extracted as described [12]. Gastrocnemius was selected due to its heterogeneous fiber type composition, and to address biological sex differences on CC onset and development by allowing comparisons to our previous study in males [12]. Total RNA concentration and purity were determined using BioTek Take3 micro-volume microplate with a BioTek Synergy HTX multi-mode plate reader (BioTek Instruments Inc., Winooski, VT), and 260/280 nm ratios and RNA concentrations were obtained. Samples were used if 260/280 ratios were of acceptable (> 2.0) quality.

### RNA Sequencing and data analysis

Complete data output and analysis can be found online for all RNA-sequencing and Pathway analysis associated data in the *Additional files*.

RNA sequencing of the gastrocnemius muscle was performed by the genomics core at Michigan State University. Briefly, libraries were prepared using Illumina TruSeq Stranded mRNA Library Preparation Kit (Illumina, San Diego, CA) per manufacturer recommendations. Libraries were divided into pools for multiplex sequencing using Illumina HiSeq 4000 flow cell in a 1 × 50 bp single read format. Base calling was completed by Illumina Real Time Analysis (RTA) v2.7.7, output was demultiplexed and converted to FASTQ files with Illumina Bcl2fastq v2.19.1. FASTQ files were organized by assigning each sample to designated groups according to condition and timepoint (PBS, 1wk, 2wk, LT, HT). Pre-alignment QA/QC read was performed to assure quality, followed by a STAR 2.7.8a index alignment – RefSeq: *mus musculus* (mouse) – mm39 GeneBank assembly. QA/QC was repeated post-alignment. Normalization was performed by using the Median ratio for DESeq2 in Partek. A filter of 30 minimum reads across all samples was applied. DESeq2 analysis with comparisons of each timepoint (1wk, 2wk, LT, HT) against control (PBS) and LT against HT was performed. Results were downloaded in text format and further organized in Excel. Cutoffs for DEGs (differentially expressed genes) were performed at 0.05 false discovery rate (FDR-adj. P-Value). Pathway analysis was performed on DEGs for each comparison through G:profiler [52] with the following settings: all results, with statistical scope, considering only annotated genes, significance threshold of g:SC threshold (0.05), and numeric IDs treated as ENTREZGene_ACC. We performed pathway analysis using ShinyGO 0.77 [53], utilizing the same KEGG pathway [54–56] settings and a background reference gene list comprised of all genes in our dataset. To verify our results, we compared them to those obtained from G:profiler.

### Comparison of RNA sequencing to MitoCarta

DEGs were cross-referenced with the Mouse MitoCarta 3.0 [28]. Cross-reference was conducted by utilizing a custom computer software provided by Kevin B. Greene.

### Comparison of RNA sequencing to male data set

A comparison of RNA-seq data from the present study with a subset of male mice from a previous study from our group [12] was performed to assess biological sex differences in response to CC. FASTQ files from male mice were uploaded to Partek, and analysis was conducted with the same parameters utilized for female mice analysis as above. Cross-referencing of DEGs of female and male was performed by custom R command provided by Dr. Aaron Caldwell. Further DESeq2 analysis was conducted on Partek by directly comparing female and male mice RNA-Seq data with the same parameters described, specifically a 2X2 comparison of biological sex (male v female) to cachexia (PBS v cachectic (4wk[male]/HT[female]).

### Statistics

For phenotypic data, a one-way ANOVA was utilized for each dependent variable. When significant F-ratios were found, differences among means were determined by Tukey’s post hoc test. For all statistical tests, the comparison-wise error (α) rate of 0.05 was adopted. Data were analyzed through GraphPad Prism (La Jolla, CA, USA). Data expressed as mean ± standard error of the mean (SEM). Data from RNA Sequencing analysis was analyzed using DESeq2 through Partek Flow. False discovery rate (FDR, reported FDR step-up adjusted *P*- value) was controlled at < 0.05.

## Supplementary Information

**Additional file 1. **Top unique to female dysregulated pathways (a), Top unique to maledysregulated pathways (b). Top 20 of each Kegg, Reactome, and WikiPathways.Adjusted *P*-value<0.05. 

**Additional file 2. **Female DESeq data output.

**Additional file 3. **Female Pathway Analysis.

**Additional file 4. **Female DEGs vs Mitocarta.

**Additional file 5. **Male DEseq data output.

**Additional file 6. **Female vs Male DESeq.

**Additional file 7. **Male Pathway Analysis.

**Additional file 8. **Female vs Male Pathway Comparison.

**Additional file 9. **Male DEGs vs Mitocarta.

**Additional file 10. **Female vs Male Mitocarta Comparison.

## Data Availability

Raw sequencing data are available in Gene Expression Omnibus: GSE222317.
