# Supplementary figures and images for: The time-course of cancer cachexia onset reveals biphasic transcriptional disruptions in female skeletal muscle distinct from males
Source: BMC Genomics. 2023 Jul 4;24:374. doi: 10.1186/s12864-023-09462-7 (PMC10318789; doi:10.1186/s12864-023-09462-7)

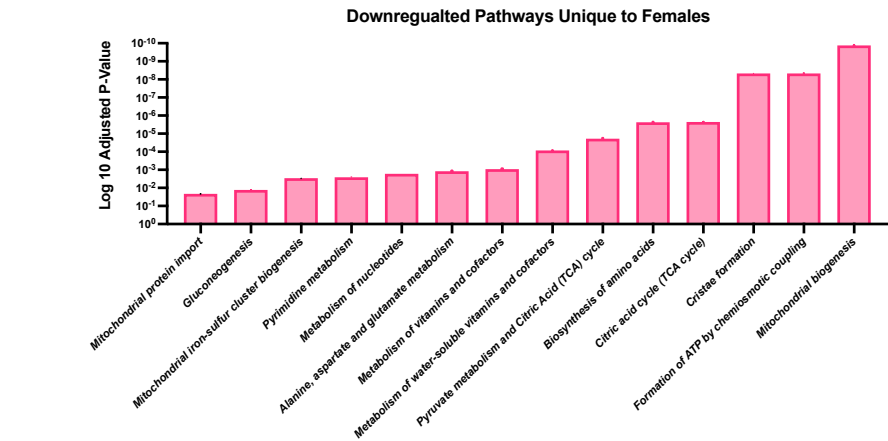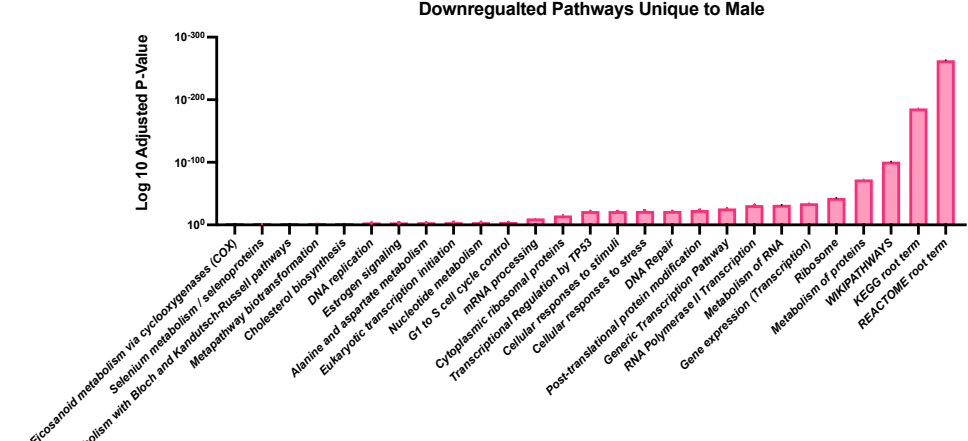

Supplement: Supplementary file 1 — Additional file 1. Top unique to female dysregulated pathways (a), Top unique to maledysregulated pathways (b). Top 20 of each Kegg, Reactome, and WikiPathways.Adjusted P-value<0.05. [file 12864_2023_9462_MOESM1_ESM.pdf]
